# Dataset for country profile and mobility analysis in the assessment of COVID-19 pandemic

**DOI:** 10.1016/j.dib.2020.105698

**Published:** 2020-05-13

**Authors:** Marcel da Câmara Ribeiro-Dantas, Gisliany Alves, Rafael B. Gomes, Leonardo C.T. Bezerra, Luciana Lima, Ivanovitch Silva

**Affiliations:** a Institut Curie, Paris, France; b Ecole Doctorale Informatique, Télécomunications et Electronique, Paris, France; c Federal University of Rio Grande do Norte, Natal, Brazil

**Keywords:** COVID-19, Social Distancing, Mobility, Health Informatics, Pandemic, Sociodemographic data

## Abstract

Understanding the COVID-19 pandemic is a multidisciplinary effort that requires a significant number of variables. This dataset comprises (i) sociodemographic characteristics, compiled from 35 datasets obtained at UN Data; (ii) mobility metrics that can assist the analysis of social distancing, from Google Community Mobility Reports and; (iii) daily counts of cases and deaths by COVID-19, from the European Centre for Disease Prevention and Control and the Johns Hopkins University Center for Systems Science and Engineering. This unified dataset ranges from February 15, 2020 to May 7, 2020, a total of 83 days, and is provided as a collection of time series for 131 countries with 192 variables. The pipeline to preprocess and generate the dataset, along with the dataset itself, are versioned with the Data Version Control tool (DVC) and are thus easily reproducible.

Specifications Table

**Table:** 

**Subject**	Infectious Diseases
**Specific subject area**	Data related to sociodemographic characteristics, community mobility variation, and epidemiological numbers related to the SARS-CoV-2 pandemic.
**Type of data**	Tab-Separated-Value (TSV)
**How data were acquired**	The original datasets were available as comma-separated values (CSV) files in the official websites of the UN Data, Google Community Mobility Reports, and the European Centre for Disease Prevention and Control, and in the GitHub repository of Johns Hopkins University Center for Systems Science and Engineering.
**Data format**	Raw Filtered
**Parameters for data collection**	We looked for datasets that could add relevant information regarding the locations that had presented occurrences of COVID-19 up to May 7, 2020. We've made sure to have data as up to date as possible, and also data related to the main questions that have been asked about the pandemic. These include social distancing and sociodemographic variables that could lead to different characteristics of the pandemic.
**Description of data collection**	Sociodemographic data for countries was downloaded from the data portal of the United Nations. Mobility data was downloaded from Google's Community Mobility Reports website. COVID-19 data was downloaded from the European Centre for Disease Prevention and Control website and from the Johns Hopkins University Center for Systems Science and Engineering GitHub repository.
**Data source location**	The dataset is hosted at DAGsHub and Mendeley Data.
**Data accessibility**	Repository name: Dataset for country profile and mobility analysis in the assessment of COVID-19 pandemic Data identification number: 10.17632/tggrsbz3bb.11 (DOI) Direct URL to data: https://data.mendeley.com/datasets/tggrsbz3bb/11 All versioned datasets, pipelines and source codes can be viewed at https://dagshub.com/mrd/DIB_COVID19_paper
**Related research article**	Ahmet Aktay et al, Google COVID-19 Community Mobility Reports: Anonymization Process Description (version 1.0), arXiv.org, arXiv:2004.04145v2.

## Value of the Data

• This dataset can be used by other researchers to apply statistical analyses and machine learning algorithms to compare the characteristics of the pandemic among different countries and try to identify characteristics that could bring new insights about the pandemic and how to fight it;
• Public managers can use this dataset to define policies. For instance, the community mobility data can be used as grounds for social distancing strategies;
• This dataset can also be used together with other datasets (such as sociodemographic and COVID-19 data on regions and subregions) to assess COVID-19 more granularly;
• The amount of data available is good enough for training, validating, and testing machine learning models; however, further expansion of the dataset would be extremely valuable.

## 1 Data Description

This dataset contains information about the COVID-19 pandemic in 131 countries. It is a single, unified dataset with 192 columns, containing several country profile characteristics, mobility data, and COVID-19 epidemiological data, ranging from February 15, 2020 to May 7, 2020. Sociodemographic data include demographic, geographic, industry, trade, government expenditure, ecologic, and political data. Mobility metrics refer to the variation in attendance to location categories when compared to a baseline period, between January 3 and February 6, before the beginning of the social distancing initiatives outside China. If a country had 15.0 for a specific day, a Thursday, for example, in the *residential* variable, this means that on that date there was a 15% positive variation in visits/staytime when compared to the median of Thursdays between January 3 and February 6 in residential areas for that country. Epidemiological data includes the daily new and accumulated number of cases of and deaths due to COVID-19, among other variables. More information can be found in the supplementary file 1 (S1 - Data Dictionary.xls).

## 2 Experimental Design, Materials, and Methods

Mobility data was downloaded from the Community Mobility Reports official website by Google [2]. To unify the remaining datasets considered, we adopted the list of countries and dates available in these reports. This decision is further justified by the impact of mobility metrics in the analysis of social distancing. The sociodemographic data for countries comes from a set of 35 datasets downloaded from UN Data, the data portal for the United Nations [1]. The daily data related to the number of new cases and new deaths by COVID-19 for countries were downloaded from the European Centre for Disease Prevention and Control (ECDC), and the dataset was downloaded as a CSV file from their official website [3]. Exceptions are Hong Kong and Réunion, that were not present in the ECDC dataset and were obtained from the Johns Hopkins University Center for Systems Science and Engineering COVID-19 repository on GitHub [4].

A pipeline was created with DVC, the Data Version Control tool, to adapt these datasets so that they could be merged, with the most updated variables, and then preprocessed afterward. DVC is used to version data and data pipelines, following the same rationale used to version source code [5]. DVC, along with Git for source code version control, allows for reproducibility so that anyone can easily reproduce our whole pipeline. The scripts for the preprocessing stage were written in the R programming language [6].

We processed the original data in three significant ways. First, we normalized country names to use them as merging key since there were minor differences in the name of the countries among the datasets. Second, we used the most up-to-date variable whenever the different datasets provided the same variable for different times. Third, new variables were engineered to enrich the dataset, which includes most of the epidemiological variables mentioned so far. For instance, we provide the date of the first COVID-19 case in each country, even for the ones that had the first case before February 15, 2020, the first date in our dataset. For countries that had the first case before February 15, we collected dates from media reports. Additional variables were computed taking as reference this per-country first case date: (i) number of days since the first case; (ii) number of days since the first death; (iii) accumulated number of cases since the first case; (iv) accumulated number of deaths since the first death; (v) date of first confirmed death, and (vi) date of first confirmed case. Finally, we also provide the latest COVID-19 lethality rate for each country.

The pipeline, datasets, and source codes are hosted at GitHub and mirrored at DAGsHub. The data is also available at Mendeley Data. In the extra folder, there are code examples on how to use the dataset written in R or Python programming languages. The preprocessed data folder contains the final dataset of this publication. Documentation that can be read as data dictionaries is provided in the documentation folder.

## Declaration of Competing Interest

The authors declare that they have no known competing financial interests or personal relationships which have, or could be perceived to have, influenced the work reported in this article.

## Appendix Supplementary materials

Image, application 1

Image, application 2
